# Effect of Recovery From Obesity on Cardiovascular Risk Factors Among Japanese Schoolchildren: The Iwata Population-Based Follow-Up Study

**DOI:** 10.2188/jea.JE20100140

**Published:** 2011-09-05

**Authors:** Katsuyasu Kouda, Yuki Fujita, Harunobu Nakamura, Hiroichi Takeuchi, Masayuki Iki

**Affiliations:** 1 Department of Public Health, Kinki University Faculty of Medicine, Osaka-Sayama, Japan; 2 Department of Health Promotion and Education, Graduate School of Human Development and Environment, Kobe University, Kobe, Japan; 3 Hamamatsu University School of Medicine, Hamamatsu, Japan

**Keywords:** blood pressure, child, cholesterol, cohort studies, obesity

## Abstract

**Background:**

The effect of recovery from obesity on cardiovascular risk factors is not well understood in Japanese children.

**Methods:**

We analyzed follow-up data from the Iwata city population-based study of schoolchildren in Japan. The Iwata Board of Education conducted health screenings of children aged 10 and 14 years. A total of 914 children aged 10 years (451 boys and 463 girls, 87.1% of all children in the city in 1997) were followed until 14 years of age and classified by pattern of obesity as Normal, Recovered, Worsened, or Persistent.

**Results:**

Of the 914 children, 111 (12%) were obese at 10 years of age. Of those children, 44 (40%) were no longer obese at 14 years (ie, Recovered). At follow-up, Recovered boys had the greatest decrease in non-HDL cholesterol (mean ± SE, −21.3 ± 3.6 mg/dL) among the 4 groups, and Recovered girls had a significantly lower level of non-HDL cholesterol (Recovered, 107.1 ± 5.4 mg/dL vs. Persistent, 126.1 ± 4.5 mg/dL). The Recovered boys also had a significantly higher level of HDL cholesterol at age 14 (Recovered, 67.2 ± 2.7 mg/dL vs. Persistent, 53.3 ± 2.1 mg/dL). In the Recovered group, 68% of children who were dyslipidemic at baseline had normal cholesterol levels at age 14. The recovery rate from dyslipidemia was significantly higher in the Recovered group (cumulative incidence rate ratio, 2.5; 95% confidence interval, 1.4–4.7) as compared with the Persistent group.

**Conclusions:**

Dyslipidemia was reversed in children who recovered from obesity. Our findings suggest that reducing obesity is beneficial to the health of Japanese schoolchildren.

## INTRODUCTION

Obesity is associated with dyslipidemia and hypertension, even in childhood.^1^^,^^2^ The relationship between changes in body fat and changes in serum lipids was reported in the 5-year longitudinal Bogalusa Heart Study of black and white children.^3^ The Minneapolis Children’s Blood Pressure Study, a population-wide longitudinal cohort study, indicated that reducing the rate of excess weight gain during childhood could reduce subsequent cardiovascular risk in adulthood.^4^ Further, the Mater-University Study of Pregnancy, a large-scale birth cohort study in Brisbane, reported that children who had a normal body mass index (BMI) at the age of 5 years but who were overweight at 14 years had elevated blood pressure; in contrast, children who were overweight at 5 years but had normal BMI at 14 years had normal blood pressure.^5^ These findings indicate that preventing and reducing obesity during childhood may provide important benefits for subsequent cardiovascular health.^6^ On the basis of evidence from a comprehensive literature search, a review study encouraged schools to take steps to prevent childhood obesity.^6^ Most school-based interventions for reducing obesity promote healthy eating and physical activity and discourage a sedentary lifestyle.^6^ In Japan, cardiovascular screening and obesity prevention programs targeting fourth to ninth grade schoolchildren have been conducted in several cities since the early 1990s.^7^^–^^9^

To complicate matters, the relationship between percent body fat and BMI is not the same for all ethnic groups.^10^ A World Health Organization expert group reported that Asian populations have a higher percentage of body fat than whites with the same BMI and that were more health risks at lower BMI levels for Asians than for whites.^11^ Similarly, ethnic differences in the relationship between body mass and cardiovascular risk were reported in a cross-sectional study of South Asian and white children in the United Kingdom,^12^ which suggests that racial differences modify the association between BMI and cardiovascular risk in childhood.

With regard to Japanese children, large-scale population-based follow-up studies have yielded few data on the relationship between obesity and cardiovascular risk.^13^^–^^15^ In addition, the effect of recovery from obesity on cardiovascular risk factors in Japanese children is not known. Therefore, in the present study, we analyzed data from the Iwata city population-based follow-up study, where schoolchildren underwent cardiovascular screening at the ages of 10 and 14 years, to evaluate the effect of recovery from obesity on cardiovascular risk factors in Japanese children.

## METHODS

### Study population

Iwata City is located in Shizuoka prefecture, which is approximately 230 km from Tokyo, Japan. An overview of the city and the cardiovascular risk status of Iwata children were previously published.^9^ Height, weight, BMI, and serum lipid levels of Iwata children were similar to the mean values of Japanese children.^9^

Our study was approved by the Ethics Committee of Kinki University School of Medicine. The target population was all fifth-grade schoolchildren (*n* = 1047) enrolled in elementary schools in Iwata City, Japan in 1997. There were no private elementary schools in Iwata City; thus, all children who lived in the city were enrolled in the 11 public schools, which were administered by the Iwata Board of Education. The Board conducted health screenings at age 10 and 14 years in 1997 and 2001. We followed a total of 914 children (451 boys and 463 girls, 87.3% of the target population) for at least 4 years until they reached the age of 14 years. Children who moved outside the school district and those with identical full names were lost to follow-up.

Subjects were divided into 4 groups: those who were not obese at 10 years or 14 years (Normal), those who were obese at 10 years but not obese at 14 years (Recovered), those who were not obese at 10 years but obese at 14 years (Worsened), and those who were obese at both ages (Persistent). In the present study, the BMI cut-off points for obesity were 19.8 kg/m^2^ and 19.9 kg/m^2^ for 10-year-old boys and girls, respectively, and 22.6 kg/m^2^ and 23.3 kg/m^2^ for 14-year-old boys and girls. These values correspond to a BMI of 25 kg/m^2^ at age 18, which was defined as overweight by the International Obesity Task Force.^16^ In Japan, obesity is defined by the Japan Society for the Study of Obesity as a BMI equal to or greater than 25.0 kg/m^2^.^17^

### Examinations

Health examinations were conducted at each school from April to June. To ensure the accuracy and precision of the tests, the same protocol was used for each examination. Measurements of height, body weight, and resting systolic blood pressure (SBP) and diastolic blood pressure (DBP) were previously described in detail.^9^ BMI was calculated as weight (kg) divided by height squared (m^2^). Total cholesterol (mg/dL) and high-density lipoprotein (HDL) cholesterol (mg/dL) were determined at a laboratory of the Shizuoka-ken Yoboigakukyokai (Shizuoka Prefecture Preventive Medicine Association, Shizuoka, Japan), as previously described,^9^ and non-HDL cholesterol (mg/dL) was calculated as total cholesterol minus HDL cholesterol. Blood test coefficients of variation were less than 4%. After the health examination, approximately 80 10-year-old children with obesity, dyslipidemia, or high blood pressure attended a 3-hour health education class conducted by the Iwata Board of Education. However, no further measures were taken.

### Statistical analysis

Data were analyzed with SAS software for Windows version 9.1 (SAS Institute Japan Ltd, Tokyo, Japan). Measurements among the 4 groups (Normal, Recovered, Worsened, and Persistent) were compared by one-way analysis of variance. Tukey’s test was used to compare the Recovered group with the other groups. A non-HDL cholesterol level greater than or equal to the 85th percentile or HDL cholesterol level less than or equal to the 15th percentile was defined as dyslipidemia. High blood pressure was defined as an SBP or DBP greater than or equal to the 85th percentile. Fisher’s exact test was used to analyze differences in recovery from and onset of cardiovascular risk factors among the 4 groups. Cumulative incidence rate ratios were calculated for comparison between the Persistent group and the other groups (cumulative incidence rate of the other groups/cumulative incidence rate of the Persistent group). A *P* value less than 0.05 was considered to indicate statistical significance.

## RESULTS

Of the 914 children, 111 (12%) were obese at the age of 10 years. Of those children, 67 (60%) were still obese at 14 years (Persistent group) and 44 (40%) were no longer obese at 14 years (Recovered group). In contrast, 41 children who were not obese at 10 years had become obese at age 14 years (Worsened group).

Table 1 shows the mean values for boys at ages 10 and 14 and the changes in mean values from age 10 to age 14. At baseline, the Recovered boys had a significantly higher level of non-HDL cholesterol as compared with the Normal boys, and there was no significant difference between the Recovered boys and the Persistent boys. During the 4-year follow-up, the Recovered boys showed the greatest decrease in non-HDL cholesterol among the 4 groups (median, −22.0 mg/dL; maximum value, 19 mg/dL; minimum value, −49.0 mg/dL). There were significant differences between the Recovered group and the Persistent group with regard to the changes that occurred. Non-HDL cholesterol in the Recovered group did not differ from that of the Normal group at the end of the follow-up period. In addition, the Recovered boys showed the highest increase in HDL cholesterol among the 4 groups and the highest level of HDL cholesterol at age 14 years, and the Recovered group differed significantly from the Persistent group.

**Table 1.** Characteristics at baseline and follow-up in Japanese school boys, by obesity pattern

	Normal (*n* = 375)	Recovered (*n* = 22)	Worsened (*n* = 19)	Persistent (*n* = 35)	*P*-value^a^
At age 10 years					
Height (cm)	137.3 ± 0.3^b^	141.9 ± 1.2	139.8 ± 1.3	143.2 ± 0.9	<0.001
Weight (kg)	31.4 ± 0.2^b^	43.2 ± 1.0	36.7 ± 1.0^b^	48.6 ± 0.8^b^	<0.001
BMI (kg/m^2^)	16.6 ± 0.1^b^	21.4 ± 0.3	18.8 ± 0.3^b^	23.6 ± 0.3^b^	<0.001
Non-HDL cholesterol (mg/dL)	105.3 ± 1.2^b^	125.5 ± 4.9	108.6 ± 5.3	125.5 ± 3.9	<0.001
HDL cholesterol (mg/dL)	67.7 ± 0.7	66.5 ± 2.8	58.3 ± 3.1	57.7 ± 2.2	<0.001
SBP (mm Hg)	111.2 ± 0.6^b^	118.1 ± 2.4	114.3 ± 2.6	117.6 ± 1.9	<0.001
DBP (mm Hg)	58.9 ± 0.4	63.2 ± 1.8	60.2 ± 1.9	60.7 ± 1.4	0.083
At age 14 years					
Height (cm)	164.0 ± 0.3	166.0 ± 1.4	165.0 ± 1.5	168.0 ± 1.1	0.007
Weight (kg)	51.1 ± 0.4^b^	57.3 ± 1.5	66.5 ± 1.6^b^	77.3 ± 1.2^b^	<0.001
BMI (kg/m^2^)	18.9 ± 0.1^b^	20.8 ± 0.4	24.4 ± 0.4^b^	27.3 ± 0.3^b^	<0.001
Non-HDL cholesterol (mg/dL)	100.7 ± 1.3	104.2 ± 5.3	115.4 ± 5.7	121.3 ± 4.2	<0.001
HDL cholesterol (mg/dL)	62.2 ± 0.6	67.2 ± 2.7	50.2 ± 2.9^b^	53.3 ± 2.1^b^	<0.001
SBP (mm Hg)	118.9 ± 0.6	120.1 ± 2.3	120.7 ± 2.4	123.4 ± 1.8	0.107
DBP (mm Hg)	63.2 ± 0.4	64.1 ± 1.7	60.5 ± 1.9	64.4 ± 1.4	0.386
Changes from 10 years to 14 years					
Height (cm)	26.7 ± 0.2^b^	24.0 ± 0.7	25.2 ± 0.8	24.8 ± 0.6	<0.001
Weight (kg)	19.7 ± 0.2^b^	14.0 ± 0.9	29.8 ± 1.0^b^	28.7 ± 0.7^b^	<0.001
BMI (kg/m^2^)	2.3 ± 0.1^b^	−0.7 ± 0.3	5.6 ± 0.3^b^	3.7 ± 0.2^b^	<0.001
Non-HDL cholesterol (mg/dL)	−4.7 ± 0.9^b^	−21.3 ± 3.6	6.8 ± 3.9^b^	−4.3 ± 2.9^b^	<0.001
HDL cholesterol (mg/dL)	−5.4 ± 0.5^c^	0.7 ± 2.2	−8.1 ± 2.4^c^	−4.4 ± 1.8	0.034
SBP (mm Hg)	7.7 ± 0.7	2.0 ± 2.7	6.4 ± 2.9	5.8 ± 2.2	0.194
DBP (mm Hg)	4.2 ± 0.5	0.9 ± 2.1	0.3 ± 2.3	3.7 ± 1.7	0.176

HDL, high-density lipoprotein; SBP, systolic blood pressure; DBP, diastolic blood pressure; BMI, body mass index.

Data are expressed as mean ± standard error.

^a^Difference among the 4 groups by one-way analysis of variance.

^b^Comparison with the Recovered group (*P* < 0.01 by Tukey’s test).

^c^Comparison with the Recovered group (*P* < 0.05 by Tukey’s test).

Table 2 shows the mean values for girls. At age 14, the Recovered girls had a significantly lower level of non-HDL cholesterol than the Persistent group.

**Table 2.** Characteristics at baseline and follow-up in Japanese school girls, by obesity pattern

	Normal (*n* = 387)	Recovered (*n* = 22)	Worsened (*n* = 22)	Persistent (*n* = 32)	*P*-value^a^
At age 10 years					
Height (cm)	139.0 ± 0.3^b^	143.7 ± 1.4	141.7 ± 1.4	143.2 ± 1.1	<0.001
Weight (kg)	32.0 ± 0.2^b^	43.2 ± 1.0	37.5 ± 1.0^b^	47.3 ± 0.8^c^	<0.001
BMI (kg/m^2^)	16.5 ± 0.1^b^	20.9 ± 0.3	18.7 ± 0.3^b^	23.0 ± 0.3^b^	<0.001
Non-HDL cholesterol (mg/dL)	106.5 ± 1.2	109.0 ± 4.8	115.7 ± 4.8	120.1 ± 4.0	0.004
HDL cholesterol (mg/dL)	65.8 ± 0.7	62.9 ± 2.8	59.4 ± 2.8	58.8 ± 2.3	0.004
SBP (mm Hg)	113.9 ± 0.6	120.2 ± 2.5	116.3 ± 2.5	122.6 ± 2.1	<0.001
DBP (mm Hg)	61.5 ± 0.4	60.6 ± 1.9	64.4 ± 1.9	64.3 ± 1.6	0.159
At age 14 years					
Height (cm)	156.2 ± 0.3	157.3 ± 1.1	155.6 ± 1.1	155.5 ± 0.9	0.590
Weight (kg)	47.7 ± 0.3^b^	53.7 ± 1.1	61.0 ± 1.1^b^	65.1 ± 1.0^b^	<0.001
BMI (kg/m^2^)	19.5 ± 0.1^b^	21.7 ± 0.4	25.2 ± 0.4^b^	26.9 ± 0.3^b^	<0.001
Non-HDL cholesterol (mg/dL)	109.1 ± 1.3	107.1 ± 5.4	125.0 ± 5.4	126.1 ± 4.5^c^	<0.001
HDL cholesterol (mg/dL)	67.1 ± 0.7	69.0 ± 2.8	62.3 ± 2.8	62.1 ± 2.3	0.058
SBP (mm Hg)	114.9 ± 0.6	118.0 ± 2.7	118.9 ± 2.7	123.8 ± 2.2	<0.001
DBP (mm Hg)	63.1 ± 0.4	65.2 ± 1.8	65.1 ± 1.8	63.0 ± 1.5	0.498
Changes from 10 years to 14 years					
Height (cm)	17.2 ± 0.2^b^	13.6 ± 1.0	14.0 ± 1.0	12.3 ± 0.8	<0.001
Weight (kg)	15.7 ± 0.2^b^	10.5 ± 0.9	23.5 ± 0.9^b^	17.8 ± 0.8^b^	<0.001
BMI (kg/m^2^)	3.1 ± 0.1^b^	0.8 ± 0.3	6.5 ± 0.3^b^	3.9 ± 0.3^b^	<0.001
Non-HDL cholesterol (mg/dL)	2.6 ± 0.9	−1.9 ± 3.7	9.3 ± 3.7	6.0 ± 3.0	0.119
HDL cholesterol (mg/dL)	1.3 ± 0.5	6.1 ± 2.2	2.9 ± 2.2	3.4 ± 1.9	0.136
SBP (mm Hg)	1.0 ± 0.7	−2.2 ± 2.9	2.6 ± 2.9	1.3 ± 2.4	0.683
DBP (mm Hg)	1.6 ± 0.5	4.5 ± 2.0	0.7 ± 2.0	−1.3 ± 1.7	0.156

HDL, high-density lipoprotein; SBP, systolic blood pressure; DBP, diastolic blood pressure; BMI, body mass index.

Data are expressed as mean ± standard error.

^a^Difference among the 4 groups by one-way analysis of variance.

^b^Comparison with the Recovered group (*P* < 0.01 by Tukey’s test).

^c^Comparison with the Recovered group (*P* < 0.05 by Tukey’s test).

Table 3 shows recovery from and onset of dyslipidemia during the 4 years. The total number of boys and girls who recovered from dyslipidemia was significantly different among the 4 groups. In boys, the cumulative incidence rate ratio of recovery from dyslipidemia was significantly higher in the Recovered group; a similar trend was seen in girls, although the difference was not significant. The total number of boys and girls with onset of dyslipidemia was significantly different among the 4 groups. For all boys and girls, the cumulative incidence rate ratio of onset of dyslipidemia was significantly lower in the Recovered group.

**Table 3.** Recovery from and onset of dyslipidemia between ages 10 and 14 years

Pattern of obesity	No. with dyslipidemia at age 10	Recovery from dyslipidemia		No. without dyslipidemia at age 10	Onset of dyslipidemia	
	
		No. recovered (%)	Cumulative incidence rate ratio (95% CI)		No. of new cases (%)	Cumulative incidence rate ratio (95% CI)
Boys						
Normal	90	40 (44.4)	1.78 (0.80, 3.93)	285	34 (11.9)	0.30 (0.15, 0.60)
Recovered	11	8 (72.7)	2.91 (1.25, 6.74)	11	0 (0.0)	— (—, —)
Worsened	8	3 (37.5)	1.50 (0.46, 4.85)	11	5 (45.5)	1.14 (0.46, 2.78)
Persistent	20	5 (25.0)	1.00	15	6 (40.0)	1.00
		0.085*			<0.001*	
Girls						
Normal	101	53 (52.5)	1.78 (0.83, 3.81)	286	53 (18.5)	0.69 (0.29, 1.66)
Recovered	8	5 (62.5)	2.13 (0.85, 5.29)	14	1 (7.1)	0.27 (0.03, 2.12)
Worsened	9	2 (22.2)	0.76 (0.18, 3.15)	13	4 (30.8)	1.15 (0.36, 3.72)
Persistent	17	5 (29.4)	1.00	15	4 (26.7)	1.00
		0.105*			0.367*	
Total						
Normal	191	93 (48.7)	1.80 (1.04, 3.12)	571	87 (15.2)	0.46 (0.27, 0.79)
Recovered	19	13 (68.4)	2.53 (1.37, 4.67)	25	1 (4.0)	0.12 (0.02, 0.87)
Worsened	17	5 (29.4)	1.09 (0.44, 2.70)	24	9 (37.5)	1.13 (0.55, 2.32)
Persistent	37	10 (27.0)	1.00	30	10 (33.3)	1.00
		<0.01*			<0.001*	

Non-high-density lipoprotein cholesterol ≥85th percentile or high-density lipoprotein cholesterol ≤15th percentile was defined as dyslipidemia.

CI, confidence interval.

*P value among the 4 groups by Fisher’s exact test.

Table 4 shows recovery from and onset of high blood pressure during the 4 years. Differences were not significant among the 4 groups for either recovery from or onset of high blood pressure.

**Table 4.** Recovery from and onset of high blood pressure between ages 10 and 14 years

Pattern of obesity	No. with high blood pressure at age 10	Recovery from high blood pressure		No. with normal blood pressure at age 10	Onset of high blood pressure	
	
		No. recovered (%)	Cumulative incidence rate ratio (95% CI)		No. of new cases (%)	Cumulative incidence rate ratio (95% CI)
Boys						
Normal	90	44 (48.9)	1.04 (0.60, 1.79)	285	69 (24.2)	0.87 (0.40, 1.89)
Recovered	10	4 (40.0)	0.85 (0.34, 2.11)	12	3 (25.0)	0.90 (0.26, 3.08)
Worsened	6	4 (66.7)	1.42 (0.66, 3.02)	13	3 (23.1)	0.83 (0.24, 2.87)
Persistent	17	8 (47.1)	1.00	18	5 (27.8)	1.00
		0.807*			0.971*	
Girls						
Normal	89	51 (57.3)	1.00 (0.62, 1.63)	298	59 (19.8)	1.19 (0.41, 3.42)
Recovered	7	6 (85.7)	1.50 (0.87, 2.59)	15	6 (40.0)	2.40 (0.72, 8.01)
Worsened	9	6 (66.7)	1.17 (0.61, 2.23)	13	4 (30.8)	1.85 (0.50, 6.88)
Persistent	14	8 (57.1)	1.00	18	3 (16.7)	1.00
		0.530*			0.202*	
Total						
Normal	179	95 (53.1)	1.03 (0.71, 1.49)	583	128 (22.0)	0.99 (0.53, 1.86)
Recovered	17	10 (58.8)	1.14 (0.68, 1.92)	27	9 (33.3)	1.50 (0.67, 3.38)
Worsened	15	10 (66.7)	1.29 (0.79, 2.11)	26	7 (26.9)	1.21 (0.50, 2.92)
Persistent	31	16 (51.6)	1.00	36	8 (22.2)	1.00
		0.764*			0.492*	

A systolic blood pressure or diastolic blood pressure ≥85th percentile was defined as high blood pressure.

CI, confidence interval.

*P value among the 4 groups by Fisher’s exact test.

## DISCUSSION

This is the first study to report the effect of recovery from obesity on cardiovascular risk factors among Japanese schoolchildren. In this Iwata population-based 4-year follow-up study, change in obesity from age 10 to age 14 was closely related to change in serum lipid levels, especially in boys. Children who were obese at 10 years but not at 14 years showed a marked improvement in serum lipids. The mean level of non-HDL cholesterol in children who recovered from obesity was similar to that of children who were not obese at either age. This suggests that recovery from obesity can normalize obesity-related dyslipidemia, even if subjects were formerly obese and had dyslipidemia. Accordingly, taking steps to reduce obesity in children and adolescents may prevent subsequent cardiovascular disease.

The present study identified sex differences in changes in serum lipids. For example, a marked decrease in non-HDL cholesterol in the Recovered group was observed in boys, but not in girls. Different growth patterns between boys and girls might be one reason for this. Previously, we reported that pubescent children who experienced growth spurts tended to have decreased serum lipids, and children who experienced less dramatic increases in height tended to have increased serum lipids.^18^ From age 10 to age 14, the mean increase in height was 24 cm in Recovered boys and 13 cm in Recovered girls in the present study. Thus, differences in vertical growth could explain some of the sex differences in changes in serum lipids.

Childhood obesity is associated with obesity in adulthood.^19^^–^^21^ In the present 4-year follow-up study, 60% of children who were obese at age 10 years were still obese at 14 years. However, 40% of children with obesity at age 10 were not obese at age 14. Previously, the Mater-University Study of Pregnancy reported that 37% of children who were overweight at the age of 5 years had a normal BMI at 14 years.^5^ The recovery rate of 40% in the present study was similar to that reported by the Mater-University Study, despite the shorter follow-up period in our study. Nevertheless, it is unclear whether the recovery rate of 40% is common in Japanese children, because there have been no large-scale community or population-based studies of Japanese children. The reason why some children recovered from obesity between age 10 and age 14 is also unclear. Different levels of physical activity between children in elementary school versus junior high school might be one explanation. Japanese children enter junior high school at age 12, and most of them join a sports club, which increases their physical activity. One comprehensive literature review has recommended that schools take steps to increase active engagement in physical activity to reduce obesity,^6^ so that being obese at 1 time point during childhood does not necessarily translate into future obesity. The present findings support the importance of programs aimed at reducing obesity in schoolchildren.

The prevalence of obese boys and girls at age 14 (BMI equal to or greater than 18-year-old equivalent, ie, 25 kg/m^2^)^16^ was 12.0% and 11.9%, respectively, in the present Iwata children and 14.9% and 11.2% in boys and girls, respectively, aged 12 to 14 years, according to the National Nutrition Survey in Japan.^22^ Furthermore, height and weight measurements of the Iwata children were consistent with the averages for Japanese children,^9^ as were serum lipid levels.^9^ The prevalence of high total cholesterol (>220 mg/dL)^23^ at age 10 was 5.8% in boys and 4.1% in girls in the present study and 4.7% in 10-year-old boys and 4.4% in 10-year-old girls in a Japanese nationwide study.^23^ Thus, the prevalences of obesity and dyslipidemia in Iwata children are similar to the averages for Japanese children.

The Iwata study has several strengths as compared with previous studies. The fact that serum cholesterol and blood pressure were repeatedly measured during childhood/adolescence is a main strength of the study, and data at both time points were obtained using identical protocols. Second, the sample size was sufficient to determine relationships between changes in obesity and changes in cardiovascular risk. Third, the follow-up rate was 87% of all children who lived in Iwata City.

A possible limitation is that we evaluated schoolchildren from only 1 city in Japan. However, a single-center study has strengths with regard to quality control, due to the absence of intercenter variation. The anthropometric values in the present study were similar to the mean values obtained from nationwide statistics of Japanese children, and the serum cholesterol levels of Iwata children were consistent with reported mean values for Japanese children from 19 prefectures who participated in a screening and management program from 1993 to 1999.^9^ The second limitation is that blood pressure was measured at school; the precision of these measurements may thus be lower than measurements obtained at medical facilities, which might explain why the cumulative incidence rate ratio of recovery from high blood pressure for the Recovered group was not significant. This may have led to an underestimation of the association between change in BMI and blood pressure.

In this Iwata population-based 4-year follow-up study, 40% of the children who were obese at age 10 were no longer obese at age 14. This recovery from obesity was associated with normalization of obesity-related dyslipidemia. Our findings suggest the importance of programs aimed at reducing childhood obesity in Japan. Further studies are needed to clarify the impact of recovery from obesity on cardiovascular risk factors in Japanese schoolchildren.
